# Retinal Vascular Resistance Significantly Correlates With Visual Acuity After 1 Year of Anti-VEGF Therapy in Central Retinal Vein Occlusion

**DOI:** 10.1167/tvst.10.11.19

**Published:** 2021-09-24

**Authors:** Makiko Matsumoto, Kiyoshi Suzuma, Fumito Akiyama, Kanako Yamada, Shiori Harada, Eiko Tsuiki, Takashi Kitaoka

**Affiliations:** 1 Department of Ophthalmology and Visual Sciences, Graduate School of Biomedical Sciences, Nagasaki University, Nagasaki, Japan; 2 Department of Ophthalmology and Visual Sciences, Graduate School of Biomedical Sciences, Kagawa University, Kagawa, Japan

**Keywords:** central retinal vein occlusion, total capillary resistance, mean blur rate, vascular endothelial growth factor, laser speckle flowgraphy

## Abstract

**Purpose:**

To investigate whether the resistivity of all retinal vessels, termed total capillary resistance (TCR), after anti-vascular endothelial growth factor (VEGF) treatment was correlated with the outcomes of patients with macular edema secondary to central retinal vein occlusion (CRVO).

**Methods:**

In total, 67 patients with nonischemic CRVO were enrolled in this retrospective observational case series. In each patient, we examined visual acuity; central retinal thickness (CRT); mean blur rate (MBR), which represents retinal blood flow velocity; and TCR. MBR and TCR were measured by laser speckle flowgraphy.

**Results:**

During the 1-year follow-up period, nine of 67 eyes (13.4%) converted to the ischemic type (converted group), whereas 58 eyes (86.6%) remained unchanged (nonischemic group). Mean CRT significantly decreased in all groups; however, the mean visual acuity significantly improved only in the nonischemic group. Mean MBR significantly increased in the nonischemic group but remained unchanged in the converted group. Mean TCR was significantly reduced in the nonischemic group but remained unchanged in the converted group. Multiple linear regression analysis revealed that MBR and TCR were the independent factors with the strongest and second strongest correlations with visual acuity after treatment, respectively.

**Conclusions:**

These findings suggest that measurements of the independent factors MBR and TCR are useful for evaluating anti-VEGF treatments in patients with CRVO.

**Translational Relevance:**

Development of clinically relevant technologies.

## Introduction

The degree of ischemia in patients with central retinal vein occlusion (CRVO) varies in accordance with both the individual and disease stage.^1^ In addition, the loss of visual function due to CRVO depends strongly on the extent of macular edema and retinal ischemia development. The outcomes of visual acuity differ greatly depending on whether it is nonischemic type or ischemic type.^1^^,^^2^ As vascular endothelial growth factor (VEGF) is the primary mediator of macular edema and retinal angiogenesis,^3^^–^^5^ anti-VEGF agents, such as aflibercept, bevacizumab, and ranibizumab, have been used in treatments for CRVO-associated macular edema.

Although intravitreal injections of anti-VEGF agents can significantly reduce macular edema and improve visual acuity,^6^^–^^10^ this approach often results in no or only a temporary therapeutic benefit, even if patients receive multiple injections.^11^^–^^14^

Previously, we evaluated mean blur rate (MBR), which reflects the retinal blood flow velocity, using a laser speckle flowgraphy (LSFG)-NAVI system (Softcare Co., Ltd., Fukuoka, Japan) in patients with CRVO.^15^^,^^16^ In addition, we reported that MBR after intravitreal bevacizumab (IVB) injection is strongly associated with the prognosis of visual acuity.^16^ In a recent study, we evaluated CRVO based on the aspect of the resistivity of all retinal vessels, termed total capillary resistance (TCR).^17^ CRVO was originally thought to be a disease caused by an increased resistance of the central retinal vein in lamina cribrosa. In CRVO, it is believed that as TCR increases MBR decreases accordingly. Therefore, based on this, we speculated that TCR would be useful for assessing the pathophysiology of CRVO. In the present study, we further used TCR to evaluate nonischemic CRVO cases and then reexamined the factors involved in the prognosis of visual acuity.

## Methods

We carried out this retrospective observational case series in accordance with the tenets of the Declaration of Helsinki and after receiving approval by the Institutional Review Board of Nagasaki University Hospital. We enrolled consecutive patients with macular edema related to CRVO in the study who had undergone anti-VEGF treatment at Nagasaki University Hospital between December 2010 and December 2018. Patients for whom we were unable to obtain proper measurements (e.g., those with cataracts with severe opacity, vitreous hemorrhage, poor mydriasis, or corneal opacity; those in which the nonperfusion area could not be accurately evaluated by fluorescein angiography), who had a history of vitreoretinal surgery, or who were classified as ischemic type or undetermined at their first visit were excluded. Before the study began, informed consent was obtained from all patients. At their first injection, we collected aqueous humor and then measured the VEGF concentration using enzyme-linked immunosorbent assays.

All patients underwent ophthalmic examinations performed by slit-lamp biomicroscopy and were assessed for best-corrected distance visual acuity with the decimal fraction, central retinal thickness (CRT) as measured by optical coherence tomography (OCT), and retinal blood flow as measured by LSFG at each visit. On their initial visit (and subsequently when deemed appropriate), all patients also underwent fluorescein angiography. The Central Retinal Vein Occlusion Study Group defines ischemic type as a case with more than 10 disc areas of nonperfusion.^18^ In line with this previous study, we classified our current patients as either the ischemic or nonischemic type. In our current study, we performed panretinal photocoagulation immediately after a case was identified as ischemic type. After the treatment, the patients were examined monthly and re-treated if the CRT was 300 µm or more. Subsequently, they were treated with a modified and extended regimen.

Sixty-seven nonischemic patients and 14 ischemic patients were evaluated at their first visit during the study period. Next, we retrospectively observed and divided the nonischemic cases into the following two groups based on the clinical course: nonischemic or converted. When evaluating these changes, fluorescein angiography was performed as needed (e.g., when visual acuity decreased, MBR decreased, bleeding increased), with the division made according to whether there were more than 10 disc areas of nonperfusion or narrower. At their first visit, nonischemic cases were classified according to whether they had converted to the ischemic type (converted group) or not (nonischemic group) on the final day of observation.

### LSFG Blood Flow Measurements

Measurements were obtained using the LSFG system. As has been previously described,^19^^,^^20^ use of the LSFG technique makes it possible to measure the optic disc blood flow. We evaluated microcirculation at the optic nerve head (ONH) by measuring the MBR of the ONH, and we evaluated total resistivity throughout all of the retinal vessels (ranging from the retinal artery, arterioles, capillaries, and venules to the central retinal vein) by measuring the TCR, as previously reported.^17^

LSFG can analyze blood flow as a series of pulsatile blood flows over several cardiac cycles for 4 seconds. As a result, LSFG can detect peak-to-peak blood flow in the cardiac cycle. To evaluate peak-to-peak blood flow using LSFG, we calculated the beat strength (BS) as being proportional to the amplitude between the maximum and minimum blood flow. The formula for calculating BS can be viewed within the patent application W0/2018/003139, Blood Flow Dynamic Imaging Diagnosis Device and Diagnosis Method (https://patentscope2.wipo.int/search/en/detail.jsf?docId=WO2018003139).^21^ TCR, which is the new parameter for the resistivity of the retinal vein, is calculated based on the ONH for CRVO using the following equation: TCR = (BS in the area of ONH)/MBR, where the MBR represents the average blood flow velocity of major vessels (arteries and veins) in the ONH. The parameter BS represents the proportional value of the peak-to-peak blood flow corresponding to the major vessels in the ONH. As a result, the TCR represents the total resistivity throughout all of the retinal vessels (including the retinal artery, arterioles, capillaries, venules, and central retinal vein).

### Retinal Thickness Analysis

CRT determinations were performed by OCT (Cirrus HD-OCT; Carl Zeiss Meditec, Jena, Germany) using the Macular Cube 512 × 128 scanning protocol, which measures the mean retinal thickness in the central 1000-µm-diameter area.

### Statistical Analysis

In this study, the primary objective was to determine the presence of a correlation between retinal blood flow levels and the outcomes among patients receiving anti-VEGF treatment for macular edema secondary to CRVO. First, we compared outcomes between the nonischemic and converted groups using the Mann–Whitney *U* test or Pearson's χ^2^ test. The mean CRT and mean MBR before and after treatment in each group were compared using a paired *t*-test. In addition, we carried out linear regression analysis to evaluate measurement of the visual acuity at 1 year after the first anti-VEGF injection, as assessed using a logarithm of the minimum angle of resolution (logMAR) chart, and other factors, as well as tests of regression. Multiple regression analysis of the 63 cases with complete data was conducted. All statistical analyses were performed using R 3.5.2 (R Foundation for Statistical Computing, Vienna, Austria). The results are expressed as the mean ± standard deviation, unless otherwise indicated. Values of *P* < 0.05 were considered to indicate statistical significance.

## Results

This study assessed a total of 67 eyes in 67 consecutive patients with nonischemic-type CRVO (36 males and 31 females; mean age, 68.4 ± 11.4 years). No patients had any prior treatment for macular edema related to CRVO. Among these 67 patients, 40, 12, and four had hypertension, diabetes mellitus, and cardiovascular disease, respectively, and 14 had no past clinical history. Of the 67 nonischemic eyes, nine (13.4%) converted to the ischemic type during the study. The mean duration from CRVO onset to the first anti-VEGF treatment was 1.1 ± 0.8 months.

Table 1 shows the characteristics of each group. No significant differences in gender, age, duration from CRVO onset to first anti-VEGF injection, VEGF concentration, number of anti-VEGF injections per year, or history were observed between the two groups.

**Table 1.** Group Characteristics (*N* = 67)

	Nonischemic Group	Converted Group	*P*	All
Number (male/female)	58 (31/27)	9 (5/4)	0.83	67 (36/31)
Age (yr), mean ± SD	68.4 ± 11.9	68.4 ± 7.9	0.98	68.4 ± 11.4
Duration from CRVO onset to first visit (mo), mean ± SD	1.0 ± 0.8	1.4 ± 1.1	0.26	1.1 ± 0.8
VEGF concentration (pg/mL), mean ± SD	312 ± 347	705 ± 1183	0.38	376 ± 571
Number of injections/yr, mean ± SD	4.2 ± 2.4	5.6 ± 1.9	0.07	4.3 ± 2.4
History, *n* (%)				
Hypertension	33 (56.9)	7 (77.8)	0.23	40 (59.7)
Diabetic mellitus	11 (19.0)	1 (11.1)	0.49	12 (17.9)
Cardiovascular disease	4 (6.9)	0 (0)	0.55	4 (6.0)

**Figure 1.**
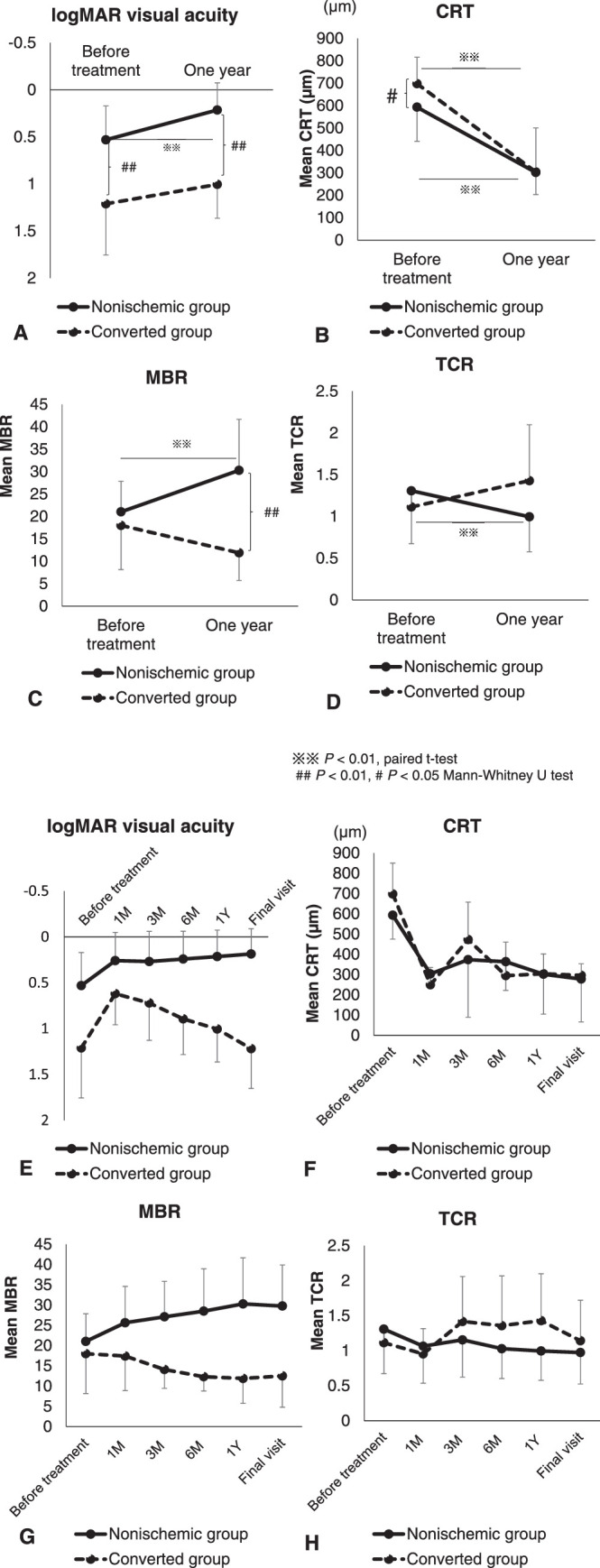
(A) Graph of changes in mean logMAR visual acuity for each group. Significant improvement in mean logMAR visual acuity was observed in the nonischemic group (before treatment: 0.53 ± 0.36 vs. 1 year after the first anti-VEGF injection: 0.21 ± 0.29; *P* < 0.01, paired *t*-test). In contrast, improvement in visual acuity was not seen in the converted group (before treatment: 1.21 ± 0.54 vs. 1 year after the first anti-VEGF injection: 1.00 ± 0.36; *P* = 0.38). A significant difference in mean logMAR visual acuity before treatment and at 1 year after the first anti-VEGF injection was found between the two groups (*P* < 0.01, Mann–Whitney *U* test). (B) Graph of changes in mean CRT for each group. A significant decrease in mean CRT was observed in the nonischemic group (before treatment: 593 ± 152 µm vs. 1 year after the first anti-VEGF injection: 302 ± 98 µm; *P* < 0.01, paired *t*-test). Similarly, in the converted group, mean CRT at 1 year after the first anti-VEGF injection was significantly lower than that observed before treatment (before treatment: 698 ± 118 µm vs. 1 year after the first anti-VEGF injection: 304 ± 197 µm; *P* < 0.01, paired *t*-test). A significant difference in CRT before treatment was observed between the two groups (*P* = 0.03, Mann–Whitney *U* test). (C) Graph of changes in mean MBR for each group. In the nonischemic group, the mean MBR significantly increased (before treatment: 21.0 ± 6.8 vs. 1 year after the first anti-VEGF injection: 30.3 ± 11.4; *P* < 0.01, paired *t*-test). In the converted group, however, MBR remained unchanged (before treatment: 18.0 ± 9.8 vs. 1 year after the first anti-VEGF injection: 11.9 ± 6.2). The differences between mean MBR values at 1 year after the first anti-VEGF injection in the nonischemic versus the converted group were statistically significant (*P* < 0.01, Mann–Whitney *U* test). (D) Graph of changes in mean TCR for each group. A significant decrease in TCR was observed in the nonischemic group after the anti-VEGF treatment (before treatment: 1.31 ± 0.63 vs. 1 year after the first anti-VEGF injection: 1.00 ± 0.42; *P* < 0.01, paired *t*-test). In the converted group, however, TCR remained unchanged after the anti-VEGF treatment (before treatment: 1.11 ± 0.23 vs. 1 year after the first anti-VEGF injection: 1.43 ± 0.67; *P* = 0.17). (E–H) Time course of changes for mean logMAR visual acuity (E), CRT (F), MBR (G), and TCR (H) in each group.

Figure 1A shows the changes in the mean logMAR visual acuity for each group. Significant improvement was observed for the mean logMAR visual acuity of the nonischemic group (before treatment: 0.53 ± 0.36 vs. 1 year after the first injection: 0.21 ± 0.29; *P* < 0.01). In contrast, improvement in the visual acuity was not seen in the converted group (before treatment: 1.21 ± 0.54 vs. 1 year after the first injection: 1.00 ± 0.36; *P* = 0.38). A significant difference in mean logMAR visual acuity was found between the two groups before treatment and at 1 year after the first injection (*P* < 0.01).

Figure 1B shows the changes in the mean CRT in each group. In the nonischemic group, a significant decrease was observed in the mean CRT (before treatment: 593 ± 152 µm vs. 1 year after the first injection: 302 ± 98 µm; *P* < 0.01). Similarly, in the converted group, mean CRT at 1 year after the first injection was significantly lower than that observed before treatment (before treatment: 698 ± 118 µm vs. 1 year after the first injection: 304 ± 197 µm; *P* < 0.01). A significant difference in CRT was observed between the two groups before treatment (*P* = 0.03).

Figure 1C shows the change in the mean MBR in each group. In the nonischemic group, mean MBR significantly increased (before treatment: 21.0 ± 6.8 vs. 1 year after the first injection: 30.3 ± 11.4; *P* < 0.01). In the converted group, however, the MBR was unchanged (before treatment: 18.0 ± 9.8 vs. 1 year after the first injection: 11.9 ± 6.2; *P* = 0.15). The differences between the mean MBR values at 1 year after the first injection in the nonischemic versus the converted group were statistically significant (*P* < 0.01). Figure 1D shows the changes in the mean TCR in each group, with a significant decrease noted for TCR in the nonischemic group after the anti-VEGF treatment (before treatment: 1.31 ± 0.63 vs. 1 year after the first injection: 1.00 ± 0.42; *P* < 0.01). In the converted group, however, TCR was unchanged after the anti-VEGF treatment (before treatment: 1.11 ± 0.23 vs. 1 year after the first injection: 1.43 ± 0.67; *P* = 0.17).

Figures 1E to 1H show the time course of changes for the mean logMAR visual acuity, CRT, MBR, and TCR in each group, respectively. The mean CRT tended to decrease after treatment in both groups (Fig. 1F). In addition, the mean TCR tended to decrease in both groups at 1 month after the first injection, with the mean visual acuity also tending to improve at 1 month after the treatment. Subsequently, the mean TCR tended to increase and the mean visual acuity tended to decrease in the converted group. In contrast, in the nonischemic group, the mean TCR remained low and the mean visual acuity remained high (Figs. 1E, 1H). With regard to MBR, the nonischemic group tended to increase, and the converted group tended to decrease (Fig. 1G).

**Figure 2.**
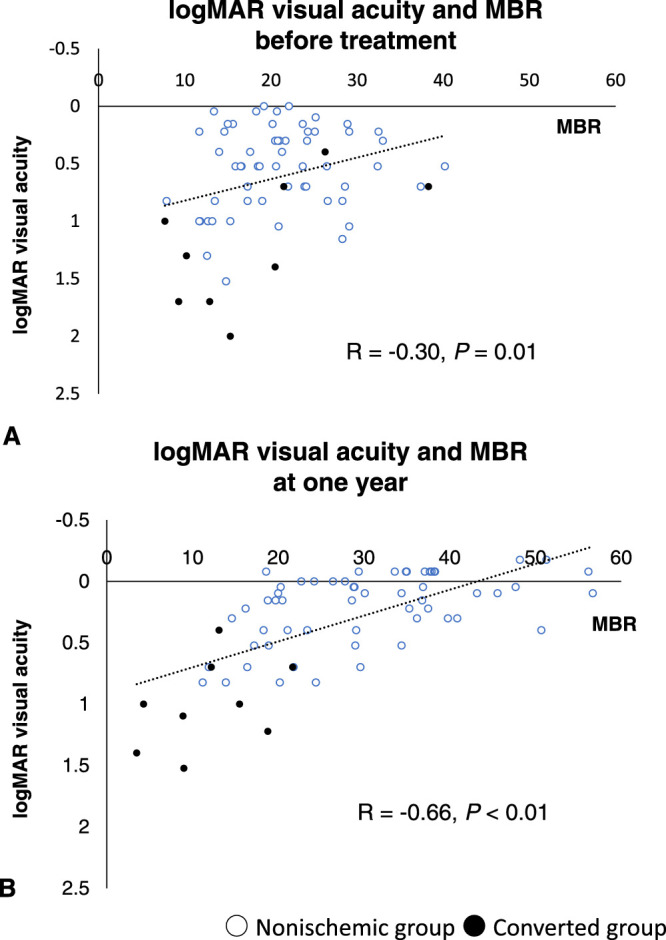
Distribution map showing logMAR visual acuity and MBR (A) before treatment and (B) at 1 year after the first treatment. A negative correlation was observed between logMAR visual acuity and MBR (A) before treatment (*R* = –0.30; *P* = 0.01) and (B) at 1 year after the first anti-VEGF injection (*R* = –0.66; *P* < 0.01).

**Figure 3.**
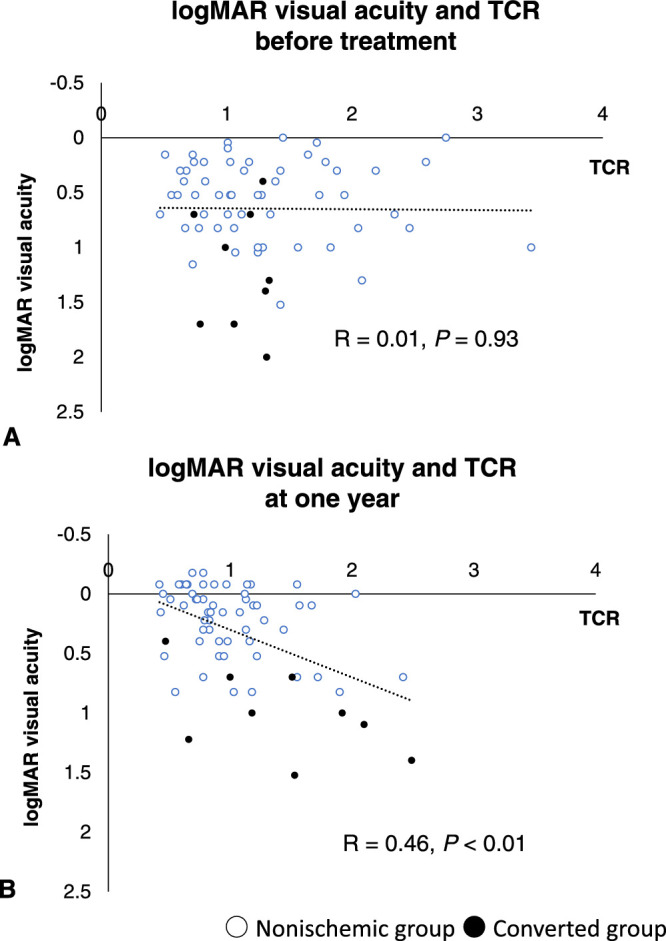
Distribution map showing logMAR visual acuity and TCR (A) before treatment and (B) at 1 year after the first treatment. (A) No correlation was found between logMAR visual acuity and TCR before treatment (*R* = 0.01; *P* = 0.93), but (B) a significant positive correlation was found at 1 year after the first anti-VEGF injection (*R* = 0.46; *P* < 0.01).

**Figure 4.**
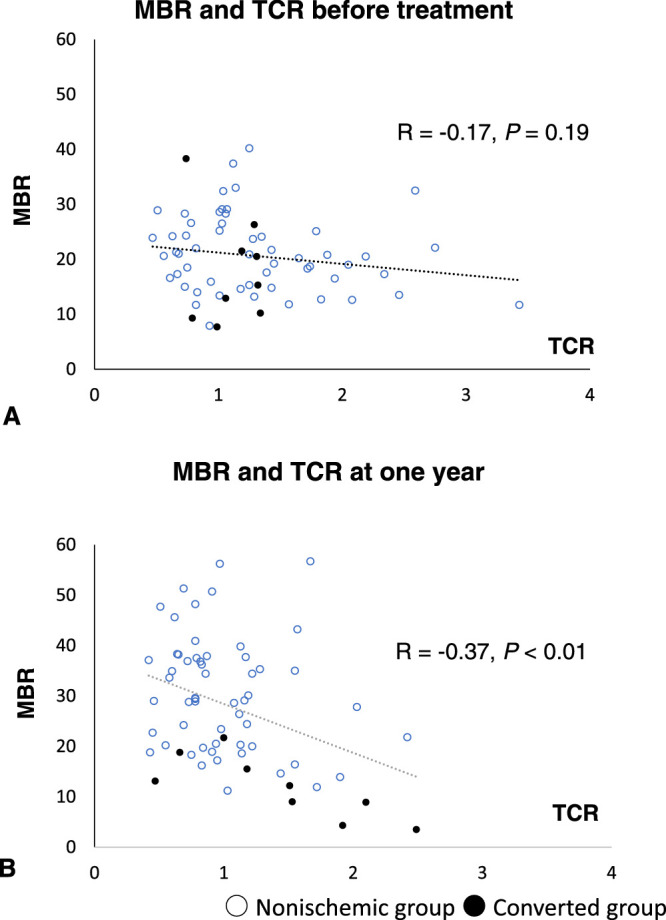
Distribution map showing MBR and TCR (A) before treatment and (B) at 1 year after the first treatment. (A) No correlation was found between MBR and TCR before treatment (*R* = 0.17; *P* = 0.19), but (B) a significant negative correlation was found at 1 year after the first anti-VEGF injection (*R* = –0.37; *P* < 0.01).

A negative correlation was found between logMAR visual acuity and MBR before treatment (*R* = –0.30; *P* = 0.01) (Fig. 2A) and at 1 year after the first injection (*R* = –0.66; *P* < 0.01) (Fig. 2B). Although no correlation was found between logMAR visual acuity and TCR (*R* = 0.01; *P* = 0.93) (Fig. 3A) before treatment, a significant positive correlation was observed at 1 year after the first injection (*R* = 0.46; *P* < 0.01) (Fig. 3B). Although a significant negative correlation was found at 1 year after the first injection (*R* = –0.37; *P* < 0.01) (Fig. 4B), no correlation between MBR and TCR was seen before treatment (*R* = –0.17; *P* = 0.19) (Fig. 4A).

Our investigation revealed significant correlations between logMAR visual acuity at 1 year after the first injection and other factors (Table 2), as well as with age (*R* = 0.29; *P* = 0.02, linear regression analysis), number of anti-VEGF injections per year (*R* = 0.29; *P* = 0.02), presence or absence of hypertension (*R* = 0.27; *P* = 0.03), CRT before treatment (*R* = 0.32; *P* < 0.01), MBR at 1 month after the first injection (*R* = –0.44; *P* < 0.01), MBR at 1 year after the first injection (*R* = –0.66; *P* < 0.01), and TCR at 1 year after the first injection (*R* = 0.46; *P* < 0.01). No statistically significant correlations were observed for the duration from CRVO onset to first visit, VEGF concentration in the aqueous humor, CRT after treatment, ocular perfusion pressure at any point, MBR before treatment, TCR before treatment, or TCR at 1 month after the first injection.

**Table 2.** Correlations With logMAR Visual Acuity After 1 Year

	Correlation Coefficient	*P*
Gender	0.008	0.95
Age	0.29	0.02^a^
Duration from CRVO onset to first visit (mo)	0.18	0.13
VEGF (pg/mL)	0.12	0.43
Number of anti-VEGF injections/yr	0.29	0.02^a^
History		
Hypertension	0.27	0.03^a^
Diabetic mellitus	–0.07	0.56
Cardiovascular disease	0.08	0.51
CRT before treatment	0.32	<0.01^b^
CRT 1 mo after first anti-VEGF injection	0.01	0.93
CRT 1 yr after first anti-VEGF injection	0.19	0.13
OPP before treatment	0.17	0.18
OPP 1 mo after first anti-VEGF injection	0.05	0.67
OPP 1 yr after first anti-VEGF injection	0.03	0.98
MBR before treatment	–0.15	0.21
MBR 1 mo after first anti-VEGF injection	–0.44	<0.01^b^
MBR 1 yr after first anti-VEGF injection	–0.66	<0.01^b^
TCR before treatment	0.04	0.73
TCR 1 mo after first anti-VEGF injection	0.13	0.32
TCR 1 yr after first anti-VEGF injection	0.46	<0.01^b^

OPP, ocular perfusion pressure.

a *P* < 0.05.

b *P* < 0.01.

We performed a multiple regression analysis with logMAR visual acuity at 1 year after the first injection as the dependent factor and gender, age, duration from CRVO onset to the first visit, CRT, MBR, and TCR as the independent factors. The multiple linear regression analysis also revealed that MBR and logMAR visual acuity at 1 year after the first injection had the strongest correlation (*T* = –5.61; *P* < 0.01) (Table 3), followed by TCR and logMAR visual acuity at 1 year after the first injection (*T* = 3.45; *P* < 0.01).

**Table 3.** Results of the Multiple Linear Regression Analysis (Dependent Factor: logMAR Visual Acuity at 1 Year After the First Anti-VEGF Injection)

	Estimate	Standard Error	*T*	*P*
Hypertension	0.15	0.087	1.70	0.09
CRT before treatment	0.26	0.088	2.98	<0.01
MBR 1 yr after first anti-VEGF injection	–0.51	0.091	–5.61	<0.01
TCR 1 yr after first anti-VEGF injection	0.32	0.094	3.45	<0.01

## Discussion

Anti-VEGF treatment has been shown to be highly effective for macular edema secondary to CRVO, but not in all cases.^11^^–^^14^^,^^22^ There were several cases of macular edema recurrence in the present study. A previous study reported that 15% and 34% of cases converted from the nonischemic to the ischemic type at 4 months and 3 years, respectively.^1^ The percentage of converted cases in this study, with a follow-up period of 12 months, was 13.4%, which could be related to the actual anti-VEGF treatment. According to the Rubeosis Anti-VEGF (RAVE) trial, VEGF blockade delayed but did not ameliorate the risk of neovascular complications.^23^ Thus, the reason why only a small number of cases converted from the nonischemic to the ischemic type in the present study might have been because the anti-VEGF therapy delayed the time to conversion to ischemia. Advanced age is a well-known risk factor for CRVO,^24^^,^^25^ as is age among patients who have received bevacizumab therapy.^11^ In the present study, a strong and significant correlation was found between age and logMAR visual acuity at 1 year after the first anti-VEGF injection (*R* = 0.29; *P* = 0.02). The results of the BRAVO and CRUISE trials indicated that initial treatment should be started immediately in patients with CRVO.^26^ In the present study, no difference in the duration from CRVO onset to the first visit was found between the two groups. In our previous study (mean follow-up period, 19.7 ± 8.4 months; treated with IVB), the number of times was significantly different between the two groups (nonischemic group: 4.3 ± 3.2 times vs. converted group: 13.0 ± 7.2 times; *P* = 0.02, Mann–Whitney *U* test).^16^ However, there was no significant difference observed for the number of anti-VEGF injections seen during the 12-month period in the present study (converted group: 5.6 ± 1.9 times vs. nonischemic group: 4.3 ± 2.4 times; *P* = 0.07). Furthermore, a strong and significant correlation was found between logMAR visual acuity at 1 year after the first injection and the number of anti-VEGF injections per year (*R* = 0.29; *P* = 0.02). These findings suggest that frequent anti-VEGF treatments do not necessarily improve the grade of ischemia. Anti-VEGF therapy has also been reported to attenuate increases in areas of nonperfusion.^8^^,^^27^^–^^29^ In contrast, the RAVE trial reported that VEGF blockade delayed but did not ameliorate the risk of neovascular complications.^23^ Therefore, this effect might be limited based on the grade of ischemia. Hypertension has long been known to be a risk factor for CRVO.^30^^,^^31^ In the present study, 40 of 67 patients (59.7%) had a history of hypertension, which was not as high as previously reported (89.2% and 89.7%).^30^ However, patients with hypertension had a poor prognosis for the visual acuity (*R* = 0.27; *P* = 0.03).

In line with previous studies,^6^^,^^7^ mean CRT significantly decreased at 1 year after the first treatment in both groups. Before treatment, although the CRT was significantly higher in the converted group, no difference was observed at 1 year after the first treatment. Post-treatment CRT was not correlated with logMAR visual acuity after 1 year, although CRT before treatment was correlated with logMAR visual acuity after 1 year (*R* = 0.32; *P* < 0.01). Although improvement in mean visual acuity was seen at 1 year after the first treatment in the nonischemic group, no significant improvement was observed in the converted group. Between the two groups, mean logMAR visual acuity was significantly poor in the converted group before and at 1 year after the first treatment. Therefore, as previously reported,^18^ poor visual acuity before treatment may result in subsequent poor visual acuity after treatment.

We previously reported that patients with a good response to IVB showed reduced CRT and increased MBR after treatment.^15^ Although a comparison of the two groups revealed no significant difference in mean MBR before treatment (nonischemic group: 21.0 ± 6.8 vs. converted group: 18.0 ± 9.8), we did find a significant difference in mean MBR at 1 year after the first injection (nonischemic group: 30.3 ± 11.4 vs. converted group: 11.9 ± 6.2; *P* < 0.01). In addition, a significant increase in MBR was seen in the nonischemic group after treatment. In contrast, MBR in the converted group remained unchanged after treatment. Regarding TCR, a significant decrease was seen in only the nonischemic group at 1 year after the first treatment (before treatment: 1.31 ± 0.63 vs. 1 year after treatment: 1.00 ± 0.42; *P* < 0.01). Based on the time course of changes observed for each index from Figures 1E to 1H, the following is suggested. The prognosis may be good in cases where the TCR decreases and the MBR increases after anti-VEGF treatment. Cases in which the TCR has decreased after anti-VEGF treatment but for which the MBR cannot be raised may result in ischemia. Thus, this suggests that it would be very useful to evaluate MBR and TCR after anti-VEGF treatment in patients with macular edema associated with CRVO. We speculate that, in the converted group, the high TCR value observed between 3 months and 1 year may be due to vascular occlusion and increases in the vascular resistance.

A significant negative correlation was found between logMAR visual acuity and MBR both before treatment (*R* = –0.30; *P* = 0.01) and at 1 year after treatment (*R* = –0.66; *P* < 0.01), with the correlation becoming stronger after treatment. Although no correlation was observed between TCR and logMAR visual acuity before treatment, a significant correlation was found at 1 year after treatment (*R* = 0.46; *P* < 0.01). Similarly, no correlation was found between MBR and TCR before treatment, whereas a significant negative correlation was found at 1 year after treatment (*R* = –0.37; *P* < 0.01).

Multiple linear regression analysis confirmed that MBR had the strongest independent correlation with visual acuity after treatment, followed by TCR. These findings suggest that outcomes in patients with CRVO can be assessed by blood flow after anti-VEGF treatment. Therefore, patients who have increased MBR and decreased TCR after anti-VEGF treatment might require only anti-VEGF therapy. However, additional treatment may be needed for patients with decreased MBR and increased TCR.

Early peripheral laser photocoagulation of a nonperfused retina has been shown to improve vision in patients with CRVO,^32^ and laser photocoagulation has been reported to increase retinal blood flow in eyes with CRVO.^33^ Therefore, we speculate that early photocoagulation might lead to better outcomes.

This retrospective study had several limitations. First, the number of patients, particularly in the converted group, was low, which could make a definitive statistical interpretation of our results difficult. Second, as this was a retrospective study, fluorescein angiography was not always repeated in all cases. As a result, it cannot be ruled out that some of the cases that were classified as being in the nonischemic group may have had an increased nonperfusion area. Third, we only undertook one approach for the blood flow measurements. Noninvasive laser Doppler instrumentation, such as LSFG, can be used to measure the absolute value of retinal blood flow.^34^^–^^37^ Therefore, different measurement methods (for example, the use of laser Doppler instrumentation) should ideally be employed when undertaking these types of studies.

In conclusion, evaluating blood flow measurements (MBR and TCR) after an anti-VEGF injection are useful in helping to determine the outcomes of the subsequent treatment strategies in patients with CRVO. Furthermore, MBR and TCR are independent factors. This study will be useful in developing clinically relevant technologies.
